# Patients’ Perspectives on Transforming Clinical Trial Participation: Large Online Vignette-based Survey

**DOI:** 10.2196/29691

**Published:** 2022-02-01

**Authors:** Van Thu Nguyen, Philippe Ravaud, Viet Thi Tran, Bridget Young, Isabelle Boutron

**Affiliations:** 1 Université de Paris Centre of Research Epidemiology and Statistics Inserm Paris France; 2 Department of Public Health, Policy and Systems Institute of Population Health University of Liverpool Liverpool United Kingdom; 3 Cochrane France Paris France; 4 Centre d'Epidémiologie Clinique Hôpital Hôtel Dieu Assistance Publique des Hôpitaux de Paris Paris France

**Keywords:** randomized controlled trial, remote trial, telemedicine, patient experience, trial participation, RCTs, participation, recruitment, patient preferences, remote medicine, pharmacological treatments

## Abstract

**Background:**

Patients’ participation is crucial to the success of randomized controlled trials (RCTs). However, recruiting and retaining patients in trials remain a challenge.

**Objective:**

This study aims to describe patients’ preferences for the organization of RCTs (visits on- site or remotely) and evaluate the potential impact of fulfilling preferences on their willingness to participate in a clinical trial.

**Methods:**

This was a vignette-based survey. Vignettes were case scenarios of real clinical trials assessing pharmacological treatments. These RCTs evaluated 6 prevalent chronic diseases (ie, osteoporosis, osteoarthritis, asthma, cardiovascular diseases, diabetes, and endometriosis). Each vignette described (1) the RCT and characteristics of the treatment tested (ie, doses, administration routes) and (2) the trial procedures and different options (on-site or remotely) for how the trial was organized for informed consent, follow-up visits, and communication of results when the trial was completed. We recruited 628 participants from ComPaRe (www.compare.aphp.fr), a French e-cohort of patients with chronic diseases. The outcomes were the participants’ preferences for the way the trial was organized (on-site or remotely) and their willingness to participate in the trial.

**Results:**

Of the 628 participants who answered the vignettes, 491 (78.2%) were female (median age 55 years), with different chronic diseases ranging from endometriosis in 59 of 491 (12%) patients to asthma in 133 of 628 (21.2%) patients. In addition, 38 (6.1%) participants wanted to provide informed consent and all trial visits on-site, 176 (28%) wished to participate in the trial entirely remotely, and 414 (65.9%) wanted to combine remote-based and hospital-based visits. Considering the trial as a whole, when the trial was organized in a way that the patients preferred, the median (Q1-Q3) likelihood of participation in the trial was 90% (80-100) versus 60% (30-80) if the trial followed the patients’ nonpreferred model. Furthermore, 256 (40.8%) patients responded to open-ended questions expressing their experience with trial participation and visits to the hospital and providing suggestions for improvement. The patients emphasized the need to personalize the way a trial is organized according to each patient’s needs and conditions.

**Conclusions:**

There was a significant diversity in the participants’ preferences. Most participants preferred hybrid organization involving both on-site and remote visits. Participants were more likely to participate in a trial organized according to their preferences.

## Introduction

Patients’ participation is crucial to the success of randomized controlled trials (RCTs). However, recruiting and retaining patients in trials remain a challenge [1,2]. For example, less than a third of RCTs funded by the National Institute for Health Research (NIHR) in the United Kingdom achieved the target sample size, and about a third of RCTs in the United States recruited less than 75% of the planned sample size [1,3]. With the increasing complexity of trial procedures, patients’ decisions to take part and remain in a trial depend not only on the potential benefits and risks of the interventions evaluated but also on the practical logistics of trials and how burdensome these are to patients [4,5]. Evidence shows that participating in RCTs can be burdensome for patients, particularly due to the ways informed consent is managed, follow-up visits organized, and trial results communicated [5,6]. Several efforts have tried to ease this burden by using technologies to allow patients to take part in a trial remotely using electronic informed consent, dispensing study drugs at patients’ homes, performing entirely web-based data collection, and conducting virtual visits via video calls [7-9]. However, the model of remote trials has not significantly succeeded in improving trial recruitment and retention and might not be suitable to all patient groups [7,10].

Information about patients’ preferences for the way a trial is organized could inform the trial planning to enhance the participation rate. However, the previous literature on patients’ willingness to participate in clinical trials mainly focused on their attitudes toward the randomization, without considering other aspects of trial organization [11]. In this study, we developed a new approach, using an online vignette-based survey to involve a large number of patients to explore their preferences on trials visits (on-site or remotely) and to evaluate the impact of fulfilling their preferences on potential participation.

## Methods

### Study Design

We conducted a vignette-based survey asking patients a set of directed questions to elicit their preferences for different ways of organizing a trial. Vignettes have traditionally been used in a number of areas, including medical training to evaluate clinical practice, and are being increasingly used in research to address topics such as identifying the best trial designs for methodological questions [12-15]. In this study, vignettes were case scenarios of real clinical trials assessing pharmacological treatments. These vignettes explained to patients what a clinical trial is and what they are expected to do when participating in the clinical trial.

This study received ethical approval from the French National Institute of Health and Medical Research Ethic Committee (IRB00003888; reference 19-580). We reported our findings following the Checklist for Reporting Results of Internet E-Surveys (CHERRIES) checklist for online surveys [16].

### Vignette Development

The vignettes were developed from existing protocols of ongoing or recently completed RCTs either published or available on clinicaltrials.gov. Two patient representatives and a steering committee comprising trialists and methodologists contributed to the development of the vignettes.

#### Clinical Trial Protocol Search

We systematically searched clinicaltrials.gov and PubMed for protocols of ongoing or recently completed (2017 onward) phase 3 RCTs evaluating pharmacological treatments. We limited the search to 6 prevalent chronic diseases: osteoporosis, osteoarthritis, asthma, cardiovascular diseases, diabetes, and endometriosis (Multimedia Appendix 1). Trials were included if they had a parallel design, at least 1 year of follow-up, and a full protocol available. We excluded trials that were exclusively on patients less than 18 years old or that were conducted exclusively in Asia, Africa, and Latin America. We also excluded trials testing treatments for secondary conditions (eg, osteoporosis induced by glucocorticoids). In total, 93 RCTs were retrieved from the search, with 18 (19%) fulfilling the inclusion criteria. One protocol was chosen for each disease, with the goal of maximizing the diversity of funding and the type of administration route across the vignettes, leading to 6 protocols being chosen for vignette development (Multimedia Appendix 2).

#### Vignette Conception

Each vignette was structured in 2 parts. The first part described the RCT and the characteristics of the treatment tested (ie, doses, administration routes). The second part described the procedures of the trial and different options of how the trial was organized for each step: (1) informed consent, (2) follow-up visits, and (3) communication of results when the trial was completed. For informed consent, the participants were asked to choose 1 of the 3 ways: (1) The trial was explained to the patients, and they signed the informed consent form at the research center; (2) information was explained via a video, and the patients electronically provided signed informed consent; and (3) the trial was explained to the patients at the research center, and they then electronically signed the informed consent form at home after getting an opportunity to discuss with their family. For follow-up visits, we provided details of the number of visits and types of tests involved in each visit. The patients were asked to choose 1 of 3 options: (1) All follow-up visits happened at the research center; (2) all follow-up visits happened at the patients’ homes, with a nurse visiting their home, and tests were conducted at a local hospital if they could not be performed at home; and (3) the patients were able to decide which visits happened at the research center and which at home. We described all logistics involved in each option (eg, contact with doctor, travel and waiting time). For communication of results, the patients were asked to choose 1 of 4 options: (1) have a face-to-face meeting with investigators, (2) have a video call with investigators, (3) receive the results by email, and (4) receive the results by post. The description of each option for informed consent and communication of results were kept unchanged across the vignettes, while the number of visits and frequency of tests performed varied according to the protocols of the real RCTs. Textbox 1 presents an example of the choices for the informed consent process for the osteoarthritis RCT. Other vignettes are provided in Multimedia Appendix 3. The vignettes were then translated into French by a professional translator. A senior researcher (author IB) and a patient representative who was a native French speaker reviewed the translation to ensure its accuracy.

**Vignette for the randomized controlled trial (RCT) of osteoarthritis.**

**1.** **Informed consent**

Before you participate in the clinical trial, the research team will explain to you the study, the new treatment, and the schedule of assessments. You will sign a consent form if you agree to participate.

Where do you want to have the information of the clinical trial explained to you and sign the consent form? Choose 1 of the 3 choices below:

**At the university hospital**

You will:

- Travel to the hospital and wait to see a study doctor.
- Meet the doctor who explains the trial to you.
- Ask your questions to the doctor.
- Sign the consent form if you agree to participate.
- Keep a copy of the consent form.

**At home using the internet**

You will:

- Stay at home.
- Watch a video online explaining the trial.
- Call the study doctor by telephone if you have questions when you want during working hours.
- Discuss with your family.
- Sign the consent form via a computer when you are ready.

A copy of the consent form will be sent to you by email or by post based on your choice.

**At the university hospital and at home**

You will:

- Travel to the hospital and wait to see a doctor.
- Meet the doctor who explains the trial to you.
- Ask your questions to the doctor.
- Return home and discuss with your family.
- Call the study doctor by telephone if you have questions when you want during working hours.
- Sign the consent form via a computer when you are ready.

A copy of the consent form will be sent to you by email or by post based on your choice.

### Participants

We recruited patients from La communauté de patients pour la recherche (ComPaRe, an ongoing cohort of patients with chronic conditions in France) [17]. The patients were adults (>18 years old) who reported having at least 1 chronic condition (defined as a condition requiring health care for at least 6 months). They helped accelerating research on their conditions by completing patient-reported outcome questionnaires, suggesting ideas for new research or participating in the analysis of research projects [18-20]. By February 2020, there were 36,000 patients participating in ComPaRe. All patients provided electronic informed consent before participating in the e-cohort. By signing this electronic informed consent form to participate in ComPaRe, they agreed to receive invitations to participate in research provided via the platform.

We sent an invitation email to all patients who reported having asthma, diabetes, endometriosis, hypercholesterolemia, osteoarthritis, or osteoporosis via the ComPaRe platform. Two reminder messages were sent to nonrespondents. We then sent the questionnaire including the vignette corresponding to the patients’ chronic diseases to patients who replied yes to the invitation email via the secure ComPaRe platform. The patients were recruited from February 12 to April 23, 2020.

### Outcomes

We evaluated the following outcomes:

- Participants’ preferences for the way a trial is organized at each step: The patients were asked to indicate their preferred choices for the following questions: *Where would you like to receive the information about the trial and sign the informed consent? Where would you like to do the follow-up visits? How do you want to receive the results of the trial?*
- Participants’ willingness to participate in the trial: The patients were asked the following questions: *If this step is performed at the hospital, how likely would you participate in the trial? If this step is performed at your home, how likely would you participate in the trial? If this step is performed at both the hospital and your home, how likely would you participate in the trial?* The patients answered each question on a scale from 0 to 100, with 0 being very unlikely and 100 being very likely.
- The patients were also invited to propose new ideas for organizing each step of the trial.

They were able to express their ideas in free text by responding to open-ended statements: *If you have an idea to improve your experience with receiving information about the trial and sign informed consent, please let us know* and *If you have an idea to improve your experience with follow-up visits, please let us know*.

### Data Analysis

Descriptive analysis was used to describe the characteristics of the patients participating in the survey and their preferences for trial organization. The analysis was performed using R software (version 4.0.2).

Analysis of the patients’ answers to open-ended questions about their ideas for how clinical trials should be organized were informed by thematic analysis. Data were imported into NVivo (QSR International) to facilitate the coding process. One researcher (author VN) performed open coding through an iterative process. A subset of responses to open-ended questions from 50 participants was coded line by line. The codes were then organized into themes to create a coding framework. This coding framework was then discussed with a senior researcher (author IB) and refined by considering the context of the data with the wider data set. The coding framework was used to analyze the remaining survey responses and refined throughout the process of analysis.

### Data Sharing

De-identified data from this study are available on request to the academic researchers who have submitted a protocol to the scientific committee of ComPaRe and signed a data use agreement.

### Patient and Public Involvement

Two patient representatives participated in the development of the vignettes.

## Results

### Study Population

We sent invitation emails to 2155 patients in the ComPaRe e-cohort, including 434 (20.14%) patients with asthma, 317 (14.71%) patients with diabetes, 480 (22.27%) patients with endometriosis, 114 (5.29%) patients with hypercholesterolemia, 276 (12.81%) patients with osteoarthritis, and 534 (24.78%) patients with osteoporosis. Of the 2155 patients, 834 (38.7%) initially responded positively to our invitations. We then sent this group the survey containing the vignettes of trials corresponding to their conditions. A total of 628 (75.3%) patients answered the vignette-based survey. Table 1 presents the demographic information of the respondents. The patients mainly lived in France (621/628, 98.9%), with age ranging from 21 to 84 years (median 55 years, IQR 44-64), and were mainly female (491, 78.2%). In addition, 427 of 628 (68%) patients lived in an urban area. Most of the patients had obtained at least a high school diploma, and 107 (17%) had experience of participating in an RCT before. Nearly 377 (60%) of the patients could reach a university hospital within 1 hour of driving from their place of residence. Multimedia Appendix 4 provides the demographic information of nonrespondents.

**Table 1:** Characteristics of patients.

Characteristic		Total (N=628)	Asthma (n=133)	Diabetes (n=83)	Endometriosis (n=59)	Hypercholesterolemia (n=76)	Osteoarthritis (n= 125)	Osteoporosis (n=152)
**Gender, n (%)**								
	Female	491 (78)	107 (80)	41 (49)	59 (100)	35 (46)	97 (78)	152 (100)
	Male	137 (22)	26 (20)	42 (51)	0	41 (54)	28 (22)	0
**Age (years), median (IQR); min-max**		55 (IQR 44-64); 21-84	45 (IQR 36-52); 22-84	54 (IQR 46-63); 26-81	38 (IQR 32-45); 21-60	61 (IQR 56-69); 25-80	57 (IQR 50-66); 26-80	60 (IQR: 55-64); 23-83
**Employment^a^, n (%)**								
	Unemployed	51 (8.1)	18 (13.5)	2 (2)	7 (12)	4 (5)	10 (8)	10 (6.6)
	Apprentice	21 (3.3)	4 (3)	3 (4)	9 (15)	0	3 (2.4)	2 (1.3)
	Employed	272 (43.3)	71 (53.4)	43 (52)	39 (66)	25 (33)	41 (32.8)	53 (34.9)
	Retired	169 (26.9)	15 (11.3)	24 (29)	0	37 (49)	41 (32.8)	52 (34.2)
	Disabled	102 (16.2)	19 (14.3)	10 (12)	4 (7)	10 (13)	28 (22.4)	31 (20.4)
	Other	12 (1.9)	5 (3.8)	1 (1)	0	0	2 (1.6)	4 (2.6)
**Highest level of education^a^, n (%)**								
	No formal diploma	14 (2.2)	4 (3)	3 (4)	1 (2)	3 (4)	2 (1.6)	1 (0.7)
	Highschool diploma	99 (15.7)	18 (13.5)	18 (22)	6 (10)	13 (17)	24 (19.2)	20 (13.2)
	Vocational training	76 (12.1)	17 (12.8)	11 (13)	4 (6.8)	11 (15)	11 (8.8)	22 (14.5)
	Undergraduate/postgraduate	435 (69.2)	93 (69.9)	49 (59)	48 (81)	49 (64)	88 (70.4)	108 (71.1)
	Other diplomas	4 (0.6)	1 (0.7)	2 (2)	0	0	0	1 (0.7)
**Living area**								
	Rural area	201 (32)	45 (33.8)	33 (40)	20 (34)	20 (26)	40 (32)	43 (28.3)
	Urban area	427 (68)	88 (66.2)	50 (60)	39 (66)	56 (74)	85 (68)	109 (71.7)
**Distance to a university hospital, n (%)**								
	<1 hour	362 (57.6)	74 (55.6)	53 (64)	35 (59)	45 (59)	80 (64)	75 (49.3)
	1-2 hours	229 (36.4)	51 (38.3)	27 (33)	20 (34)	26 (34)	37 (29.6)	68 (44.7)
	2-5 hours	37 (5.9)	8 (6)	3 (3)	4 (7)	5 (7)	8 (6.4)	9 (5.9)
**Previous participation in a trial, n (%)**								
	Yes	106 (16.9)	26 (19.5)	16 (19)	4 (7)	14 (18)	19 (15.2)	27 (17.7)
	No	522 (83.1)	107 (80.5)	67 (81)	55 (93)	62 (82)	106 (84.8)	125 (82.3)

^a^One participant did not answer this question.

### Patients’ Preferences for the Way a Trial Is Organized

All patients answered the vignette of the trial testing treatment for their conditions. For the informed consent process, 311 (49.5%) patients indicated that they preferred to be given information about the trials and sign the consent form at home over the internet, while 239 (38.1%) patients preferred the 2-step approach: having information about the trial explained at the hospital and signing the consent form at home.

Regarding follow-up visits, 251 (39.9%) patients wished to have all follow-up visits at home and 254 (40.4%) patients preferred the combination of both on-site visits at research centers and home-based visits with the possibility to arrange the visits according to their choices. Only 122 (19.4%) patients chose to have all follow-up visits at the hospital.

Most of the respondents (276/628, 43.9%) wished to have an in-person meeting with research investigators when receiving the results of the trials, 192 (30.6%) chose to receive a summary of results by email, and 126 (20.1%) preferred a video call with research investigators (Table 2). Overall preferences were consistent across conditions. However, for the informed consent process, 33 of 59 (56%) patients with endometriosis preferred the trial to be explained to them at the hospital and to sign the informed consent form at home. Patients with hypercholesterolemia were the only group in which most patients chose to receive trial results by mail (33/76, 43%), while the other groups wished to meet a research investigator in person (Table 2).

**Table 2:** Patients’ choices of trial organization.

Choice			Total (N=628)		Asthma (n=133)		Diabetes (n=83)		Endometriosis (n=59)		Hypercholesterolemia (n=76)		Osteoarthritis (n=125)		Osteoporosis (n=152)
**Informed consent, n (%)**															
	At home	311 (49.5)		73 (54.9)		39 (47)		19 (32)		47 (62)		58 (46.4)		75 (49.3)	
	At hospital and at home	239 (38.1)		32 (24.1)		40 (48)		33 (56)		22 (29)		40 (32)		62 (40.8)	
	At hospital	78 (12.4)		18 (13.5)		4 (5)		7 (12)		7 (9)		27 (21.6)		15 (9.9)	
**Follow-up visits^a^, n (%)**															
	By choices	254 (40.4)		51 (38.3)		29 (35)		28 (48)		23 (30)		61 (48.8)		62 (40.8)	
	At home	251 (39.9)		58 (43.6)		42 (51)		19 (32)		41 (54)		30 (24)		61 (40.1)	
	At hospital	122 (19.4)		23 (17.3)		12 (15)		12 (20)		12 (16)		34 (27.2)		29 (19.1)	
**Receive results, n (%)**															
	Meeting a doctor at the hospital	275 (43.8)		58 (43.6)		39 (47)		32 (54)		24 (32)		62 (49.6)		60 (39.5)	
	Video call with a doctor	126 (20.1)		30 (22.6)		19 (23)		16 (27)		16 (21)		19 (15.2)		26 (17.1)	
	By mail	192 (30.6)		38 (28.6)		25 (30)		9 (15)		33 (43)		38 (30.4)		49 (32.2)	
	By post	34 (5.4)		7 (5.3)		0		2 (3)		3 (4)		6 (4.8)		16 (10.5)	

^a^One participant did not answer this question.

**Figure 1:**
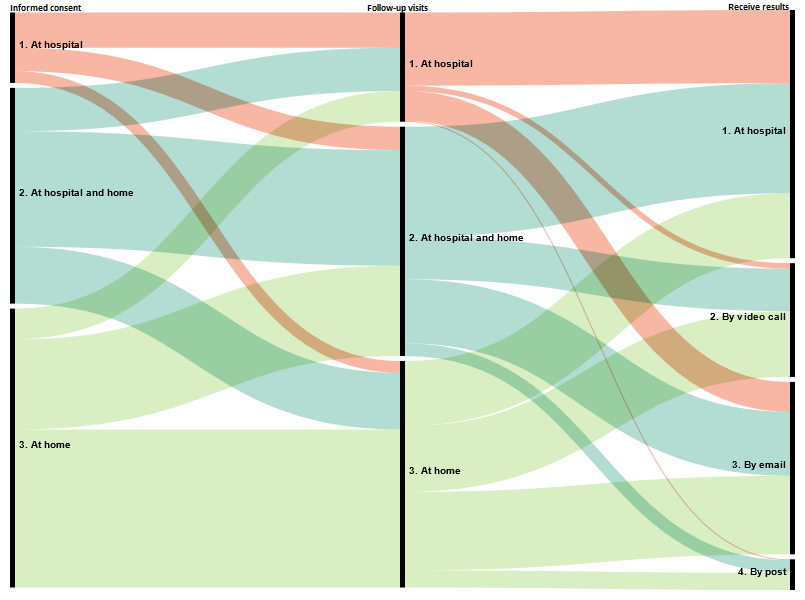
Diversity of patients’ preferences for the way a trial is organized. This alluvial diagram presents patients’ choices for each step of trial participation. The streams connecting the columns present the proportion of patients selecting each option in each column. For example, the red streams connecting “Informed consent” and “Follow-up” present the proportion of patients deciding to have follow-up visits at the hospital (n=39, 50%), at both the hospital and home (n=26, 33%), or at home (n=13, 17%) among all patients who decided to provide informed consent at the hospital (n=78). The red streams connecting “Follow-up” and “Results communication” present the proportion of patients deciding to receive the results at the hospital (n=81, 66.9%), by video call (n=6, 4.9%), by email (n=33, 27.3%), and by post (n=1, 0.8%) among all patients who decided to have all follow-up visits at the hospital (n=121).

Figure 1 illustrates the diversity of the patients’ preferences regarding trial participation as a whole. For example, of 311 (49.5%) patients who wished to have the informed consent process at home, more than half (175/311, 56.3%) chose to have all follow-up visits at home. Of these 175, 73 (41.7%) wished to receive trial results by mail, 55 (31.4%) wished to be informed about trial results via a video call, and 31 (17.7%) preferred to meet a research investigator in person.

### Patients’ Willingness to Participate in a Trial According to Their Preferences for Trial Organization

**Figure 2:**
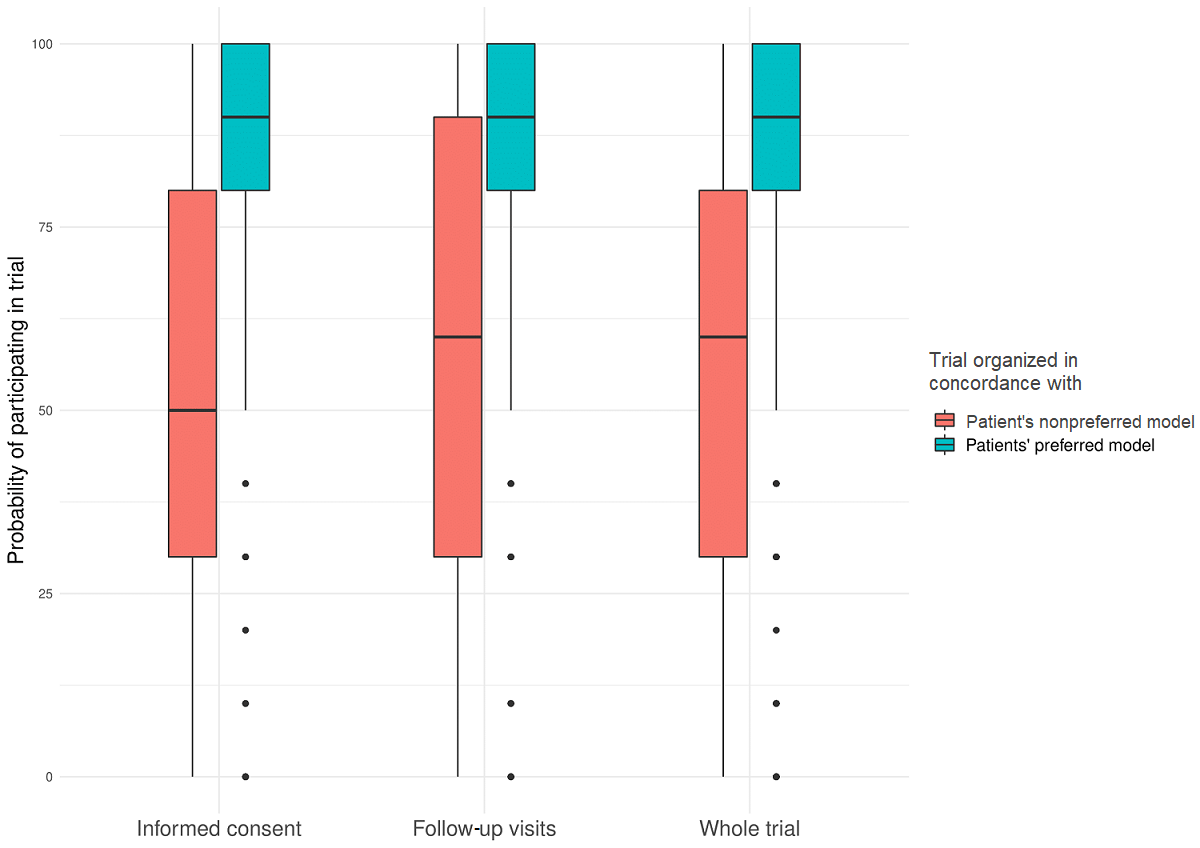
Probability of participating when a trial is performed in accordance with patients’ preferences.

When the informed consent process was organized in a way that the patients preferred, the median likelihood to participate in the trial was 90% (IQR 80%-100%) versus 50% (IQR 30%-80%) if the informed consent process followed the patients’ nonpreferred model. Similarly, the median likelihood to participate in a trial if follow-up visits followed the patients’ preferred model was 90% (IQR 80%-100%) versus 60% (IQR 30%-90%) if trials were set up according to the patients’ nonpreferred model. Considering the trial as a whole, when the trial was organized in a way that the patients preferred, the median likelihood of participation in the trial was 90% (IQR 80%-100%) versus 60% (IQR 30%-80%) if the trial followed the patients’ nonpreferred model. See Figure 2.

### Patients’ Suggestions to Improve the Way a Trial Is Organized

Of 628 patients, 256 (40.8%) responded to at least 1 open-ended question, expressing their experience with trial participation and visits at the hospital and providing suggestions for improvement. Patients emphasized the need to personalize the way a trial is organized according to each patient’s needs and conditions.

I think you cannot generalize, but for each clinical trial, the patient must be given a choice of how to participate. This depends mainly on the distance between home and hospital and of course whether the person has a professional activity or not. The way of participating could be proposed to the patient at the same time as the consent and the patient will then be in control of whether he or she can and wants to participate.

Patient #125 with asthma

### Patients’ Propositions to Improve Trial Visits at the Hospital

The patients suggested changes to the logistical organization of hospital visits to reduce waiting time and also suggested that flexible appointment times be offered to suit the individual situations of patients (Textbox 2).

**Patients’ proposals to improve visits.**

**1. Keep appointment times and reduce waiting time.**

Make sure that appointments with doctors or ECG radiography departments are on time.

Patient #41 with osteoarthritis

It all depends on the location of the hospital and how easy it is to get there by public transport (I don't drive). On the other hand, please respect the appointment times very strictly.

Patient #5 with osteoporosis preferring home-based visits

**2. Arrange a reception dedicated to trial participants.**

Make sure that appointments with doctors or ECG radiography departments are on time, without going through the general reception of the hospital . . . In order for me to participate in a study, the “logistics” must be as fluid as possible and outside the traditional care circuit in terms of administration and waiting time.

Patient #41 with osteoarthritis preferring combination of hospital and home-based visits

**3. Provide flexibility of appointment time.**

Having the possibility to have intelligent appointments, to have all examination and tests in the morning or in the afternoon or from 10:00 to 15:00, for example . . . this allows fragile, sick, and tired people to take time and take care of their health, when they come from far away or when they have difficulty to move. It is important to be able to organize according to our conditions.

Patient #13 with diabetes preferring combination of hospital and home-based visits

I would be willing to go to the hospital without any worries, but I do not want this to be done during my working hours as it should not be the concern of my employer.

Patient #65 with asthma preferring combination of hospital and home-based visits

**4. Combine follow-up visits with routine care visits.**

Should we combine the visit with the examination and radiography for osteoarthritis?

Patient #97 with osteoarthritis preferring combination of hospital and home-based visits

**5. Reimburse transportation fees and provide free parking.**

The fee of transportation and parking should be reimbursed for traveling to and parking at the research center.

Patient #35 with osteoarthritis preferring combination of hospital and home-based visits

### Patients’ Proposals Regarding Remote Trial Visits

The patients highlighted the advantages of participating in a trial from home over the internet as a solution to reduce travel, save time, and avoid disruption of their work. They, nevertheless, indicated that human contact is important and an in-person conversation with research investigators would help reassure them when making decisions related to trial participation.

In the context of a clinical trial, a contact with a real human is important. The internet does not transmit the emotion. [. . .] It is reassuring [in] the hospital setting. They (doctors) can see my condition, and I also feel that I am a stakeholder and an actor of my own decisions when having a human in front of me.

Patient #73 with osteoarthritis preferring hospital-based visits

One patient also explained that she wanted to keep her home as a private place for rest and recreation.

My home is a place of conviviality, rest, or recreation. I do not want my home to become a place of care. I already have auto-injections. I prefer to go to the doctor, in a center of care, even if that seems more constraining.

Patient #4 with asthma preferring combination of hospital and home-based visits

Several patients suggested that researchers should foresee measurements to ensure the accuracy of tests and data collected outside the context of the hospital, which might influence the quality of research. Further, they also reminded researchers about the fact that not all patients would have access to equipment for video calls with doctors and that their internet connection might be unstable.

The patients also suggested that trial investigators could collaborate with local hospitals and laboratories to organize visits and examinations, which would reduce travel time, while maintaining the quality of data collected. The patients also spoke of the possibility to involve their primary care doctors in the trials, which would help them feel more reassured when participating in trials (Textbox 3).

**Patients’ suggestions regarding remote trial visits.**

**1. Involve local hospitals and health care providers for follow-up visits.**

I participated in a clinical trial. The appointments with the doctor took place at the hospital. The biological tests between appointments at the hospital were done at a laboratory near my home. I appreciated this organization.

Patient #56 with asthma preferring hospital-based visits

To not wait too long at the hospital, and to be able to do the visits at a hospital nearby to reduce the travel time.

Patient #123 with asthma preferring combination of hospital and home-based visits

**2. Involve primary care doctors for informed consent and follow-up visits.**

Another suggestion is to involve the primary care doctor as an intermediary to explain the study.

Patient #82 with osteoarthritis preferring hospital-based visits

To involve the primary care doctor to avoid a part of the travel to the hospital.

Patient #51 with endometriosis preferring hospital-based visits

Follow-up of the trial by primary care doctor and nurse for usual blood examination in close contact with the research team of the university hospital.

Patient #37 with hypercholesterolemia preferring home-based visits

**3. Ensure close contact with investigators.**

Contact by video call rather than by telephone.

Patient #55 with diabetes preferring home-based visits

It would be good to be able to exchange email with the doctor.

Patient #63 with diabetes preferring combination of hospital and home-based visits

**4. Provide equipment suitable to patients’ conditions.**

I have a computer with a large screen, but my osteoarthritis makes me suffer so much that I cannot sit upright for more than 5 minutes. I can use [the] telephone for a video call, and I can lie down, although it will be less effective for communication.

Patient #54 with osteoarthritis preferring hospital-based visits

I would like to have home-based visits, but I am not sure to have a webcam with my computer.

Patient #6 with hypercholesterolemia preferring combination of hospital and home-based visits

**5. Apply technology to reduce the burden of data collection.**

Plan (or use an existing one) an application with file sending via email for patients already doing PeakFlow follow-ups if this can replace or complement the certain spirometry (to avoid sending an IDE at home).

Patient #115 with asthma preferring home-based visits

## Discussion

### Summary of Findings

In this study, we used a vignette-based survey to solicit patients’ preferences for the way RCTs are organized from a large group of patients. We created 6 vignettes based on protocols of real clinical trials. In total, 628 patients with different chronic diseases, ranging from 59 (9.4%) patients with endometriosis to 133 (21.2%) patients with asthma, shared their preferences. Our results highlight the diversity of the preferences and show that if trials are planned according to patients’ preferred choices, the likelihood of participating in trials could increase by 30%.

### Implications

Our results showed the desire of patients to move from the one-size-fits-all approach of trial participation and tailor the way trials are organized to better suit each patient’s condition, such as severity of their diseases, employment status, and distance to the hospital. By allowing flexibility in the way patients participate in a trial, patients who are underrepresented due to barriers, such as employment, income, and distance to hospitals, might be enabled to take part, thus increasing external validity [21,22]. The COVID-19 pandemic has created challenges for clinical trials but also created opportunities to significantly change how trials are conducted. Many clinical trials have adapted to recruit patients online and remotely perform trial visits [23]. For example, the Pragmatic Evaluation of Events And Benefits of Lipid-lowering in Older Adults (PREVENTABLE) trial shipped medications to patients’ homes and cognitive assessment was performed at the patient’s home by trained research staff [24]. A trial evaluating fluvoxamine to treat COVID-19 was conducted entirely remotely. Patients were recruited online and signed the e-consent form, and data were collected via mail or telephone [25]. These examples show the feasibility of remote trials as an alternative to overcome certain barriers to trial participation. Further research is needed to assess the validity of data collected in a setting outside the research center [26,27]. Nevertheless, other barriers, such as understanding of complex concepts related to clinical trial participation (eg, randomization) or concerns about adverse effects of tested treatments, might not be resolved by remote trials [24]. Thus, discussion to address the concerns of participants and identify the best approach for their participation in trials is necessary.

The patients in our sample also expressed the need to be informed about trial results, preferably during a discussion with a doctor, to get a chance to bring up questions and these being answered directly. This desire is in line with efforts to enhance transparency of trial results for which funders are striving. Further, informing patients about trial results helps them understand the significance of their contribution to science and could encourage them to participate again in future studies [28].

Our study also shows that vignette-based surveys are a useful and innovative design to incorporate perspectives of a large number of patients and public members in research. The vignette is an effective way to communicate the complex concept of clinical trials to patients. In the development of the vignettes for this study, we explained the process and practicalities of taking part in a trial, such as the distance of travel, types and number of examination tests, and the total amount of time for each visit. We cocreated the vignettes with patient representatives to ensure that the structure and language used were comprehensible to the patients. Vignette-based surveys allow patients to express their opinions and ideas without pressure from other stakeholders [29-32]. Vignette-based surveys could also be used to discover patients’ perspectives on other aspects of trial design, such as their preferences for comparators, outcomes, and study design.

### Limitations

Our study had some limitations. We recruited patients from a patient e-cohort; thus patients in our sample had more experience with the use of the internet and participating in research. In France, 77% of the population has smartphones and 72% of the population has access to the internet [33]. In our study, 427 of 628 (67.9%) patients lived in urban areas and 364 (57.9%) had access to a university hospital in less than 1 hour, which is similar to the French population, with 54.6% of the population living in urban areas (ie, areas with more than 50,000 inhabitants) [34]. Additionally, the majority of patients (621/628, 98.9%) lived in France; thus their experience with clinical trial participation might be different from that of patients living in other countries in order to be able to generalize the study results. Nevertheless, this study could be adapted to other languages and disseminated to international patient communities. Lastly, due to the hypothetical nature of the vignettes, we cannot exclude the fact that patients might make a different decision in a real-life situation.

### Conclusion

We used a vignette-based survey, a new approach, to solicit preferences and ideas to improve RCT organization from a large number of patients. The patients emphasized the need to transform the current one-size-fits-all approach of clinical trial participation.

## Appendix

Multimedia Appendix 1 Search strategy for trial protocols.

Multimedia Appendix 2 Trial protocols selected for vignette development.

Multimedia Appendix 3 Example of a vignette.

Multimedia Appendix 4 Nonrespondents' characteristics.

## Abbreviations

CHERRIES: Checklist for Reporting Results of Internet E-Surveys
ComPaRe: La communauté de patients pour la recherche
PREVENTABLE: Pragmatic Evaluation of Events And Benefits of Lipid-lowering in Older Adults
RCT: randomized controlled trial
