# Supplementary material for: Patients’ Perspectives on Transforming Clinical Trial Participation: Large Online Vignette-based Survey
Source: J Med Internet Res. 2022 Feb 1;24(2):e29691. doi: 10.2196/29691 (PMC8848233; doi:10.2196/29691)
Supplement: Multimedia Appendix 1 [file jmir_v24i2e29691_app1.docx]

Appendix 1. Protocol search strategy

| **Search strategy on clinicaltrials.gov** | **Search date** |
| --- | --- |
| Osteoporosis, phase 3, study protocol | 26 August 2019 |
| Osteoarthritis, phase 3, study protocol | 27 August 2019 |
| Cardiovascular disease, phase 3, study protocol | 27 August 2019 |
| **Search on PubMed** |  |
| ((asthma AND LANCET AND randomised controlled trial)) AND ("2019/01/01"[Date - Publication]: "3000"[Date - Publication]) | 13 November 2019 |
| ((diabetes AND NEJM AND randomised controlled trial)) AND ("2016/01/01"[Date - Publication]: "3000"[Date - Publication]) | 27 February 2020 |
| ((endometriosis AND NEJM AND randomised controlled trial)) AND ("2016/01/01"[Date - Publication]: "3000"[Date - Publication]) | 27 February 2020 |
