# Supplementary material for: Patients’ Perspectives on Transforming Clinical Trial Participation: Large Online Vignette-based Survey
Source: J Med Internet Res. 2022 Feb 1;24(2):e29691. doi: 10.2196/29691 (PMC8848233; doi:10.2196/29691)
Supplement: Multimedia Appendix 2 [file jmir_v24i2e29691_app2.docx]

Appendix 2. Protocols selected for vignettes development.

| Trial title | Route of administration of treatment |
| --- | --- |
| Study to Determine the Efficacy and Safety of Romosozumab in the Treatment of Postmenopausal Women With Osteoporosis | Subcutaneous injection |
| Study of the Analgesic Efficacy and Safety of Subcutaneous Tanezumab in Subjects With Osteoarthritis of the Hip or Knee. | Subcutaneous injection |
| RCT of the efficacy and safety of an ICS/ LABA reliever therapy regimen in asthma | Inhalation |
| REVEAL: Randomized EValuation of the Effects of Anacetrapib Through Lipid-modification | Oral |
| Empagliflozin and progression of kidney disease in type 2 diabetes | Oral |
| Treatment of endometriosis-associated pain with elagolix, an oral GnRH antagonist. | Oral |
