# Supplementary material for: Patients’ Perspectives on Transforming Clinical Trial Participation: Large Online Vignette-based Survey
Source: J Med Internet Res. 2022 Feb 1;24(2):e29691. doi: 10.2196/29691 (PMC8848233; doi:10.2196/29691)
Supplement: Multimedia Appendix 3 [file jmir_v24i2e29691_app3.docx]

Help us to accelerate research on Cardiovascular disease!

What are clinical trials?

Clinical trials assess if new medicines for cardiovascular disease are safe and effective. With results from clinical trials, you can receive effective treatment and avoid treatments which are ineffective and harmful.

What are the challenges of participating in clinical trials?

By their nature, clinical trials require patients to attend more assessments in addition to their normal doctors’ appointments, which means more travel and time commitment from patients and their care givers.

The goal of the REMOTE project

In the REMOTE project, our goal is to make participation in clinical trials more convenient for patients and their care givers with less travel and time commitment.

What can you do to help us?

We are asking you to please complete a short questionnaire (15min). This project received ethical approval from French National Institute of Health and Medical Research (INSERM) reference number 19-580. No identifying data will be collected. All data will be stored securely at University Paris Descartes. The data can be shared with academic researchers who will have to submit a protocol and sign a data sharing agreement.

Please confirm that you agree to participate in the REMOTE project

The clinical trial

Imagine that you or one of your family members have high cholesterol level in blood which leads to high risk of cardiovascular diseases.

There is a clinical trial which is testing a new treatment to reduce cholesterol level in blood. The new treatment will be taken orally once a day with a meal. The clinical trial will last for four years.

The trial takes place at the university hospital which is two-hour drive from your home. When participating in the trial, you will meet a doctor, receive treatment and do blood tests at the university hospital.

There is another option to participate in the trial remotely from your home, communicate with the doctor via a computer, receive treatment at home and do blood tests at a local laboratory.

Which way of participating in the trial would be more suitable for you? By answering this question, you will help us to create a better way to organize clinical trial.

1. Informed consent

Before you participate in a clinical trial, the research team will explain to you the study, the new treatment and schedule of assessments. You will sign a consent form if you agree to participate.

Where do you want to have the information of the clinical trial explained to you and sign the consent form?

| **At the university hospital** You will:   - Travel to the hospital, wait to see a study doctor - Meet the doctor who explains the trial to you - Ask your questions to the doctor - Sign the consent form if you agree to participate OR go home to discuss with your family and return to sign the consent when you are ready. - Keep a copy of the consent form. |
| --- |
| **At home using internet**  You will:   - Stay at home - Watch a video online explaining the trial - Call a study doctor by telephone if you have questions when you want during working hours - Discuss with your family - Sign the consent form via a computer when you are ready - A copy of the consent form will be sent to you by email or by post based on your choice |
| **At the university hospital and at home**  You will:   - Travel to the hospital, wait to see a doctor - Meet the doctor who explains the trial to you - Ask your questions to the doctor - Return home and discuss with your family - Call the study doctor by telephone if you have questions when you want during working hours - Sign the consent form via a computer when you are ready - A copy of the consent form will be sent to you by email or by post based on your choice |

If this step is implemented at the hospital, how likely would you participate in the trial?

| 0  Very unlikely |  | 100  Very likely |
| --- | --- | --- |

If this step is implemented at your home, how likely would you participate in the trial?

| 0  Very unlikely |  | 100  Very likely |
| --- | --- | --- |

If this step is implemented at the hospital and your home, how likely would you participate in the trial?

| 0  Very unlikely |  | 100  Very likely |
| --- | --- | --- |

1. Clinical trial visits

During four years of the clinical trial, you have a total of 10 follow-up visits to assess how well you are doing with the new treatment. You will have a visit every 6 months.

- In the first and the last visits, you will have a health check, answer a questionnaire, have blood tests and a urine test.
- In the other visits, you will have a health check, answer a questionnaire, and have blood tests.

Where do you want to do the follow-up visits?

| **All visits will be at the university hospital**  In each visit, you will:   - Travel to the hospital and wait for the doctor - Meet the doctor, have a health check - Complete a questionnaire with the doctor - Have blood tests and a urine test as planned - Each visit will take around half a day |
| --- |
| **All visits will be at your home**  In each visit, you will:   - Stay at home - Have a video call with the doctor if you need to - Complete a questionnaire online by yourself - A study nurse will visit your home to do blood tests and a urine test based on your schedule. |
| **Visit will be at the university hospital or at your home based on your choice**  This implies:   - One week before the scheduled visit, a study nurse will call you to confirm if you want to do the visit at the hospital or at home. The study nurse will arrange the visits according to your choice. |

If this step is implemented at the hospital, how likely would you participate in the trial?

| 0  Very unlikely |  | 100  Very likely |
| --- | --- | --- |

If this step is implemented at your home, how likely would you participate in the trial?

| 0  Very unlikely |  | 100  Very likely |
| --- | --- | --- |

If this step is implemented at the hospital and your home, how likely would you participate in the trial?

| 0  Very unlikely |  | 100  Very likely |
| --- | --- | --- |

## Receiving the results of the clinical trial

Your participation in the clinical trial lasts one year. But the clinical trial will only come to an end when the last patient has had all their trial visits, which might be several months after you finish your participation.

When the trial is completed, researchers will inform you about the final results of all other patients who participated in the clinical trial.

How do you want to receive the results of the trial?

- Have a face-to-face meeting with a research team member at the university hospital who will explain the results to you
- Have a video call at home with a research team member from the university hospital who will explain the results to you
- Receive written summary of the results by post
- Receive summary of the results via email
