# Supplementary material for: Patients’ Perspectives on Transforming Clinical Trial Participation: Large Online Vignette-based Survey
Source: J Med Internet Res. 2022 Feb 1;24(2):e29691. doi: 10.2196/29691 (PMC8848233; doi:10.2196/29691)
Supplement: Multimedia Appendix 4 [file jmir_v24i2e29691_app4.docx]

Appendix 4. Demographic information of non-respondents

|  | Non-respondents to invitation emails  (n = 1321) | Non-respondents to the vignette survey  (n = 206) |
| --- | --- | --- |
| Female | 1114 (84%) | 171 (83%) |
| Age | 46 (IQR: 34 – 59) [18 – 85] | 54 (IQR: 37 – 64) [22 – 84] |
| Employment* | | |
| Unemployed | 142 (11%) | 15 (7%) |
| Apprentice | 78 (6%) | 6 (3%) |
| Employed | 703 (53%) | 97 (47%) |
| Retired | 224 (17%) | 52 (25%) |
| Disabled | 135 (10%) | 31 (15%) |
| Other | 38 (3%) | 5 (2%) |
| Highest level of education* | | |
| No formal diploma | 24 (2%) | 5 (2%) |
| Highschool diploma | 256 (19%) | 28 (14%) |
| Vocational training | 185 (14%) | 23 (11%) |
| Undergraduate and postgraduate | 843 (64%) | 149 (72%) |
| Other diplomas | 14 (11%) | 1 (0.4%) |
| * one missing data | | |
